# Energy drinks: Getting wings but at what health cost?

**DOI:** 10.12669/pjms.306.5396

**Published:** 2014

**Authors:** Nahla Khamis Ibrahim, Rahila Iftikhar

**Affiliations:** 1 Nahla Khamis Ibrahim, MBBCh, MPH, Dr.PH, DHPE.Professor of Epidemiology & Public Health, Family & Community Medicine Department, King Abdulaziz University, Jeddah, Saudi Arabia.Professor of Epidemiology at the High Institute of Public Health, Alexandria University, Egypt.; 2 Rahila Iftikhar, FCPS, MRCGP. Assistant Professor, Family & Community Medicine Department, King Abdulaziz University, Jeddah, Saudi Arabia.

**Keywords:** Adverse health effects, Energy drinks, Soft drinks, Caffeine

## Abstract

Energy drink consumption represents a global public health problem, especially among adolescents and young adults. The consumption of energy drinks has seen a substantial increase during the past few decades, especially in the Western and Asian countries. Although manufacturers of energy drinks claim that these beverages are beneficial in that they can boost energy, physical performance, and improve cognitive performance, there is insufficient scientific evidence to support these claims. The known and unknown pharmacology of the constituents of energy drinks, supplemented with reports of toxicity, raise concern for the potentially severe adverse events linked with energy drink use. Limited numbers of reviews have been published on this important subject..The aim of this review was to identify the major ingredients in energy drinks and to delineate the adverse effects related to their consumption.

***Methods:*** Electronic databases of PubMed, Clinical Key, and Google and Cochrane library were extensively searched for energy drink articles. More than hundred articles were reviewed, scrutinized and critically appraised and the most relevant forty articles were used

***Conclusion:*** Energy drinks & its ingredients are potentially dangerous to many aspects of health. Measures should be taken to improve awareness among adolescents and their parents regarding the potential hazards of energy drinks. Furthermore, the sale of energy drinks on college and university campuses and to adolescents below 16 years should be prohibited.

## INTRODUCTION

‘Energy drinks’ are fortified beverages with added dietary supplements. They differ from soft or sport drinks in that they contain higher levels of caffeine in addition to sugars and other dietary supplements.^1^^,^^2^ Producers of energy drinks claim that these beverages contain natural ingredients that increase energy, attention, and improve sports performance and concentration time; however, health professionals are concerned about the adverse effects associated with these products.^3^^-^^6^

Little comprehensive literature reviews were done to illustrate the adverse health effects of energy drink, or to link these adverse effects with the recommendations and guidelines established by the International Organizations for limitation of its use in certain ages. In this context, it becomes critical to have such a deep review that can contribute towards illustration of rational of usage. So, such reviews are urgently needed

The aim of this review was to identify the major ingredients in energy drinks and to delineate the adverse effects & different recommendations related to their consumption.

## METHODS

The electronic databases PubMed, Clinical Key, and Google were searched using the terms “energy drink”, “energy drink toxicity”, “energy drink harmful effects and caffeine”, “gurana”, and “obesity and energy drink”. The Cochrane website was also searched using the key word “caffeine”. Searches were limited to articles published in English. More than hundred articles were reviewed, scrutinized and critically appraised and the most relevant forty articles were used

Articles that were funded by energy drink companies and those that included athletes were excluded from this analysis. Ethical approval was taken.

***Extent of use: ***The first energy drink was introduced in Austria in 1987 while it was launched in the United States (US) in 1997.^7^ Since then, the consumption of energy drinks has increased dramatically although they are twice as expensive as traditional soft drinks. The main consumers of energy drinks are aged 18-34 years.^8^ Like soft drinks, energy drinks are available in most grocery stores, making it easy for consumers to choose them over soft drinks.^9^

In the United States (US), nearly 200 new brands of energy drinks were launched between 2006 and 2007,^1^ and in 2004, 1.5 billion cans of Red Bull were sold in 2011.^7^ A similar increase in energy drink consumption has been reported in many countries worldwide. It was found that energy drinks are available to buy in > 140 countries, and half of the consumers of these drinks consisted of children, adolescents and young adults.^10^

***Ingestion Recommendations: ***Even though energy drinks are highly marketed, there is no strong evidence that supports their use. In 2007, the American Institute of Medicine published a report for recommending healthier eating environment for children and adolescents. Among its recommendation was to restrict carbonated, fortified, or flavored waters, restriction of sports drinks to use by athletes, prohibition of energy drink use, even for athletes & prohibition of sale of caffeinated products in school.^11^ The International Sport Society also recognizes that energy drinks contain many ingredients which need further studies to demonstrate their safety and potential harmful effects.^12^

There is limited evidence that energy drinks may cause weight loss but higher consumption of these drinks may facilitate weight gain. So, it is also recommended that children and adolescents should not consume energy drinks without parental permission.^12^ Current recommendations from Health Canada, 2013, stipulate that the daily caffeine intake for children younger should not be greater than 2.5 mg/kg of body weight.^13^

**Possible adverse effects of energy drinks & its ingredients:**

***Cardiovascular effects:*** Caffeine ingredient in energy drink is a known ergogenic substance that increases the heart rate and blood pressure. It binds to adenosine receptors on heart muscle cells, which initiates a second messenger system with cyclic adenosine monophosphate within the cells, mimicking the effects of epinephrine.^14^ Cardiovascular adverse effects such as tachycardia and arrthymia typically arise when > 200 mg of caffeine are ingested.^1^ In one report, Worthley et al.^15^ tested 50 young men and women one hour before and one after the participants ingested 250 mL of a sugar-free drink that contained approximately 80 mg of caffeine. They found that the mean arterial pressure of the participants increased by 13.7% compared to a 0.3% change in the controls. On the contrary, they found that endothelial function decreased; however, they could not accurately identify the constituent in energy drinks that produced this effect and suggested that because elevated glucose levels were associated with endothelial dysfunction and impaired platelet function, the observed effects were possibly due to the glucuronolactone in energy drinks. Prior findings demonstrated that the consumption of one cup of caffeinated coffee acutely hindered flow-mediated dilation in the brachial artery of healthy individuals, while nitroglycerin-induced vasodilatation was unaffected, implying impaired endothelium dependent vasodilatation.^16^

Caffeine has also been shown to adversely affect arterial stiffness. In their report, Mahmud and Feely^17^ showed that the consumption of caffeinated coffee, but not decaffeinated coffee, acutely increased aortic stiffness in healthy individuals.

Myocardial infarction has also been reported as an adverse effect of caffeine.^18^ In one retrospective study, Klatsky et al.^19^ reported an increased risk for cardiovascular heart disease in persons who consumed more than 6 cups of coffee per day; however, this risk was only increased for persons who were former smokers. Kabagambe et al.^20^ conducted a case-control study in Costa Rica and demonstrated presence of an association between caffeine intake and nonfatal myocardial infarction. Results of multivariate analysis revealed that the Population-Attributable Risk (PAR) was 12.8% (95% confidence interval, 5.9–25.7%). In addition, one retrospective study showed that among patients with confirmed cardiovascular heart disease, heavy coffee consumption (> 10 cups daily) was associated with a significant increase in the risk of sudden cardiac arrest.^21^ On the other hand, in a prospective study conducted on post-myocardial infarction patients, it was found that there was no association between moderate coffee intake and cardiovascular events after controlling for confounding factors.^22^

***Obesity and non alcoholic fatty liver disease: ***A balanced diet provides enough carbohydrate for an active individual and energy drinks should not be used to substitute water in between meals. Moreover, the liquid form of carbohydrate provides no satiety, and it can increase the total amount of solid food intake, and thereby nullify the effects of exercise.^23^^,^^24^ A previous review reported that greater sugar-sweetened beverage—including soft drinks, fruit drinks, energy, and vitamin water drinks — consumption is positively associated with overweight and obesity. Findings from a recent study also indicate that sugar-containing beverages increase the risk for diabetes mellitus and cardio-metabolic diseases, as the beta cells become unable to secrete sufficient insulin to maintain normal blood glucose when the body is exposed to excesses of simple sugars over long periods.^25^

***Neurological: ***Frequent consumption of caffeine may negatively impact cognition in general and perpetual memory and learning specifically.^26^ Furthermore, moderate doses of caffeine impair motor skill and may be an inadequate substitute for memory enhancements or daytime sleep.

In 2002 Smith^27^ published a report illustrated that caffeine at a dose of 300 mg can increase anxiety and tension. At a dose of 400 mg caffeine increases anxiety; especially when paired with a stressful task. Other authors have attempted to investigate caffeine-associated panic attacks in patients who suffer from anxiety. Based on their findings, Nardi et al.^28^ suggested that patients with panic disorder demonstrated increased sensitivity to low doses of caffeine when compared with depressive patients. After the administration of caffeine, patients with panic disorders had an increase in subject-related anxiety, nervousness, fear, and tremors.

Hallucinatory experiences are reportedly more likely to occur in individuals who consume > 300 mg of coffee (approximately 7 cups) per day than in those who consume low levels of caffeine (one to three cups per day).^29^ The hallucinatory experiences, as suggested by some authors,^30^ may be due to caffeine augmenting the physiological effects of stress, as evidenced by the release of cortisol during stressful periods, following recent caffeine ingestion. The additional upsurge of cortisol after caffeine use may link the higher tendency for individuals to hallucinate with caffeine consumption.^31^

Some reports suggested that energy drinks can precipitate first-onset seizure and contribute to stroke.^32^ However, there is insufficient evidence to support these findings.

***Musculoskeletal and renal:*** Caffeine is rarely cited as a potential cause of rhabdomyolysis with one report describes a case of rhabdomyolysis due to caffeine overdose.^33^ Energy drinks, can cause hypokalemia because of their diuretic effect can also cause high levels of creatinine kinase and renal impairment.^34^ Persons who do not typically consume large amounts of caffeine (in energy drink) may experience increased diuresis when they consume energy drinks. Hence, energy drinks have a net dehydrating effect.^35^

***Dental Erosion: ***Dental erosion is a well-known adverse effect of high carbonated drinks.^36^ Recent findings have demonstrated a significant association between energy drinks and dental erosion.^37^ In one meta-analysis, it was reported that the consumption of soft drinks was associated with approximately a 2.4-fold risk of dental erosion. The demineralization potential of energy beverages is principally due to their low pH.^38^

***Effects of mixing energy drinks with other beverages: ***In one community survey,^39^ it was reported that 6% of adolescents mixed alcohol with energy drinks. Energy drinks were mixed or consumed simultaneously with alcohol during parties. In some cases premixed alcoholic drinks were consumed. The practice of mixing energy drinks with alcohol has been linked consistently to harmful drinking behaviors. For example, it was found that consumers of premixed alcoholic beverages were more likely to drink high volumes of alcohol per drinking session.^40^

## RECOMMENDATIONS

The potential serious adverse effects of energy drink consumption prompt us to recommend that at the clinical level, health care providers should inquire about the misuse of energy drinks at every clinical encounter, mainly with adolescents. A history of energy drink consumption should be comprehensive, for example, the amount of cans consumed, volume, duration, type of energy drink consumed, and drink-mixing habits. History-taking should also include the use of tobacco (*Shisha* in Saudi Arabia), adverse effects and emergency visits related to caffeinated drink use. Clinicians should screen for energy drink use in the same manner as they would in cases of substance misuse. Medical counseling and education may be useful.

At the community level, measures should be taken to improve awareness among adolescents, as well as their parents, regarding the potential hazards of consuming energy drinks. School authorities should implement programs that teach healthy dietary habits. Furthermore, local authorities should prohibit the sale of energy drinks on college and university campuses, and fitness clubs and coaches should discourage their use. The sale of energy drinks should also be prohibited to adolescents below 16 years. 

Future studies should be performed to assess the long term complications associated with the consumption and drinking pattern of energy drinks, especially in the Middle East; these studies should also evaluate the financial cost of importing energy drinks. To improve toxicity surveillance, the healthcare sector should encourage the documentation of adverse events and emergency visits related to caffeine intoxication after energy drink consumption.

Regulations regarding marketing, in both print and electronic media, and sales are to be reinforced on the bases of current scientific knowledge. Consumption should be based on appropriate research and the approval of food regulatory bodies and drug regulating authorities. Finally, religious leaders and scholars should give a verdict about the use of energy drinks, as there are concerns that they may contain alcohol, which is strictly forbidden in Islam. In addition, the other ingredients in energy drinks, when combined with alcohol, can have an addictive potential. In summary, clinicians and health policy makers have the shared responsibility to educate the youth, as they are our future.
